# How Are Sociodemographic, Health, Psychological, and Cognitive Factors Associated with Dementia Worry? An Online Survey Study among Israeli and Australian Laypeople

**DOI:** 10.3390/ijerph191811313

**Published:** 2022-09-08

**Authors:** Perla Werner, Sarang Kim

**Affiliations:** 1 Department of Community Mental Health, University of Haifa, Mt. Carmel, Haifa 3498838, Israel; 2 School of Psychology, University of New South Wales, Kensington 2052, Australia; 3 Neuroscience Research Australia, Randwick 2031, Australia

**Keywords:** dementia, worry, culture, emotions, Israel, Australia

## Abstract

Background: Dementia worry is a widespread phenomenon and the most common emotional reaction elicited by the threat of developing dementia in the future. The cultural factors of dementia worry have not been examined widely, although dementia can be perceived differently between cultures and lead to varying levels of dementia worry. The purpose of this study was to examine the level of dementia worry and factors associated with it cross-nationally in Israel and Australia. Methods: A cross-sectional, online survey was conducted with two age-matched adult samples (447 participants in Israel and 290 in Australia). The primary outcome measure was the 12-item Dementia Worry Scale. Results: Israeli participants (mean age = 42.5 years; 50.1% female) reported statistically significantly (*p* < 0.05) higher levels of concern about developing dementia in comparison to Australian participants (mean age = 43.7 years; 49.8% female). Increased ageism and increased perceptions about the likelihood of developing dementia were the most important factors associated with increased worry. Conclusions: Our findings suggest that country may not be the best criterion to assess cultural differences and should be accompanied by the participants’ assessment of their cultural tendencies. Our study also stresses the importance of conceptualizing and assessing affective and cognitive aspects of dementia worry, as people from different cultures might perceive dementia worry cognitively differently but affectively similar and vice-versa.

## 1. Introduction

### 1.1. Dementia Worry

Dementia worry is the most common emotional reaction elicited by the threat of developing dementia in the future. As the older population increases worldwide, and the number of people with dementia rises accordingly [1], efforts to understand the meaning and reduce the negative consequences of this phenomenon proliferate.

However, as noted in a recent review of 45 studies [2], the investigation of dementia worry is characterized by conceptual and methodological problems. Conceptually, there is no clear and consistent definition of the construct, and terms such as dementia worry, dementia fear, and dementia anxiety are used interchangeably. Methodologically, structured and validated measures to assess the construct are lacking. These limitations are worrisome, especially as the same review demonstrated that levels of dementia worry reported in the studies ranged from low to high, depending on the samples and measures used.

Investigations of factors associated with dementia worry are also scarce. The most important and consistent factors described in the literature have included sociodemographic characteristics (increased age, female gender, and exposure to dementia), health characteristics (decreased general and cognitive health), psychological characteristics (increased depression and distress), and cognitive characteristics (increased dementia knowledge, and stigmatic beliefs) [3,4,5,6,7]. However, there may be other understudied factors associated with dementia worry, such as culture. The cultural aspects of dementia worry, however, remain largely unknown. Indeed, to the best of our knowledge, to date, only one study has explored and characterized dementia worry cross-culturally. The results of this qualitative study—based on a sample of 130 laypeople comprising people with mild neurocognitive disorder and their relatives in Israel and Germany—revealed similarities as well as differences in the meaning attributed to dementia worry and in its triggering and coping mechanisms across cultures [8]. The findings of this pioneering study stress the importance of extending research in the area of dementia worry and culture, especially among non-affected people.

### 1.2. Dementia and Culture

Culture means shared ideas, beliefs, values, and traditions that influence how we see and act in the world. Hofstede’s individualism/collectivism dimension is the most common measure used to examine cultural values across societies [9]. Individualistic cultures place a high value on autonomy and independence and encourage individuals to care mostly for themselves. Collectivistic cultures value interdependence and encourage individuals to see themselves as part of in-groups or collectivities.

Cultural norms, values, and beliefs help create shared knowledge within a group about diseases. Thus, dementia can be perceived differently in different cultures. Evidence shows that dementia care varies between collectivistic and individualistic societies. Family members carry more of the caregiving burden for someone with dementia in collectivistic cultures, whereas institutions or professionals tend to do most of the caregiving in individualistic societies [10]. Therefore, people in different cultures will likely have different levels of exposure and experience with dementia, affecting their perceptions and their levels of worry.

### 1.3. Emotions and Culture

As stated, worry or fear is a basic human emotion common to all cultures. However, different cultural values may lead to different emotional responses [11]. Thus, the way worry is felt and expressed is not only biologically determined but also influenced by cultural context [12]. Cross-cultural studies have shown that emotions and their expression, valence, and regulation vary among countries with different cultural values [13,14,15].

Although few in number, some studies have indeed examined the emotions of fear or anxiety toward different targets among individualistic and collectivistic cultures, but these studies have yielded inconsistent results. For example, whereas some reported positive associations between collectivistic values and fear [16,17,18,19], others reported just the opposite [20]. Moreover, as stated, studies examining these associations with dementia worry are remarkably lacking.

### 1.4. Objective and Hypotheses of the Current Study

The purpose of this study was to examine levels of dementia worry and the factors associated with it cross-culturally in Israel and Australia. The reason these countries were selected is that despite their both being Western, multicultural societies, they differ in several demographic and cultural characteristics. Demographically, compared to Australia, Israel is a younger country. Data from the World Bank [21] shows that 12% of the population in Israel is aged 65 and above, compared to 16% in Australia. Accordingly, in terms of the prevalence of dementia, there are 10 and 14 people per 1000 people living with dementia in Israel and Australia, respectively [22]. As these numbers are expected to increase in future years, both countries have initiated national strategic programs to improve the quality of life and care provided to people living with dementia and their caregivers, reduce the risk of dementia, and increase awareness about the disease [23,24]. Culturally, Australia is highly individualistic, whereas Israel is only moderately so, according to Hofstede’s ranking of countries [25].

Based on the similarities and differences between Israel and Australia, we expected to find differences in the level of dementia worry reported by participants in these two countries. However, based on the inconsistencies reported above regarding the associations between cultural dimensions and emotions, we did not formulate a direction for this hypothesis. Finally, based on the literature described above, we hypothesized that increased age, female gender, worse subjective health, higher levels of dementia knowledge, higher perceptions of the likelihood of developing dementia, as well as higher levels of ageism and dementia stigma, would be significantly associated with increased levels of dementia worry.

## 2. Materials and Methods

### 2.1. Design and Procedure

We conducted a cross-sectional survey with two age-matched samples (447 participants in Israel and 290 in Australia) using a similarly structured survey. Sample size calculation was undertaken using G*power (version 3.1.9.4) [26]). To detect a medium effect size (0.15), with a 5% α and 95% power in multiple linear regression, at least 194 participants were required. Being 18 years of age or older and fluent in the language used in the questionnaire were the only criteria to participate in the survey, and no exclusion criteria were applied.

In Israel, data were collected online by a major Israeli internet panel company (*p*-value), which maintains panels of potential participants for the entire Israeli population. Panelists who adhered to the inclusion criteria were invited to participate in the online survey from 20 June to 24 June 2018. Once quotas by gender, age, and region were reached for each parameter, the survey was closed. In Australia, a web address for the online survey was distributed to a nationally representative sample by Qualtrics. Quotas were set up to match the census distribution on age and gender, and potential participants were allowed to take part in the survey from 15 June to 19 June 2018.

### 2.2. Instruments

#### 2.2.1. Dependent Variable

*Dementia Worry*: The Dementia Worry Scale (DWS) [27] was used to assess dementia worry. This is a well-validated measure, including 12 items rated on a Likert-type scale (1—“not at all typical of me” to 5—“very typical of me”). The DWS was not available in Hebrew and was, therefore, translated back and forth by two independent researchers. The Hebrew version of the DWS exhibited excellent internal consistency (Cronbach’s alpha = 0.92 in Israel). We calculated an overall index by averaging the scale’s items.

#### 2.2.2. Independent Variables

*Dementia Stigma*: We used nine items to assess the cognitive, emotional, and behavioral dimensions of public stigma. Example items included: “People with dementia should be put into a nursing home”; “I am afraid of people with dementia”; and “I will try to keep distance from people with dementia.” [28]. Items were rated from 1—“strongly disagree” to 9—“strongly agree”. The best internal reliability in both samples (Cronbach’s alpha = 0.64 and 0.76 for Israel and Australia, respectively) was obtained by averaging 6 out of the 9 items.

*Subjective Knowledge about Dementia*: One item (rated from 1—“not much at all” to 5—“very much”) was used to assess how much participants estimated they know about dementia [29].

*Ageism*: The 18-item Hebrew version and the original 20-item scale [30] were used among the Israeli sample and the Australian sample, respectively. Twelve common items (rated from 1—“strongly disagree” to 4—“strongly agree”) from the Fraboni Scale of Ageism [31] were used in analyses. An overall index of the mean score of the items was calculated, with high scores reflecting higher levels of ageism. The overall scale demonstrated moderate internal reliability in both countries (Cronbach’s alphas = 0.68 in the Israeli sample and 0.63 in the Australian sample).

*Familiarity with Dementia*: One item was used to know whether participants knew someone with dementia or not [29].

*Likelihood of Developing Dementia*: A single item (rated 1—“not at all likely” to 5—“very likely”) was used to assess to what extent participants think they are likely to develop dementia [29].

*Background Information:* Information was collected regarding participants’ gender (male/female), age, and ethnicity (i.e., majority = Jewish for Israel, and British and European for Australia; or minority = Arab for Israel and all other ethnic groups for Australia), and regarding their health and financial status (from 1 to 5, with higher scores reflecting better statuses).

### 2.3. Statistical Analyses

A comparison of the characteristics of the Israeli and Australian samples, as well as of the main study variables, was conducted using independent samples *t*-tests and chi-squared tests. To examine the associations between the study variables and dementia worry among the total sample, a multiple linear regression was conducted. In the first step, background variables that were related to dementia worry were entered as control variables (gender, ethnicity, and familiarity with dementia). In the second step, subjective knowledge of dementia and ageism were entered. In the third step, country was entered. Finally, in the fourth step, interactions between country and each of the independent variables were entered in a stepwise manner. All continuous variables were standardized. Dementia worry and dementia stigma were log-transformed due to positively skewed distributions, and all continuous variables were standardized.

## 3. Results

### 3.1. Participants’ Background Characteristics

As can be observed in Table 1, no statistically significant differences were found between Israel and Australia in their gender and age composition: In both countries, the sample was almost equally divided between male and female participants, and the mean age was around 43 years. However, a statistically significantly larger proportion of the Australian sample reported themselves to be part of an ethnic majority (84%) compared to the Israeli sample (78%), indicating that the Israeli sample had more participants who defined themselves as belonging to ethnic minorities. Lastly, Israeli participants perceived themselves as having better health and financial status than did Australian participants.

### 3.2. Comparison between Israeli and Australian Samples in Study Variables

A comparison between the Israeli and Australian age-matched samples (Table 1) showed several differences between the countries regarding the study’s main variables. Regarding dementia worry, Israeli participants reported statistically significantly (*p* < 0.05) higher levels of concern about developing dementia in comparison to Australian participants. Similarly, they reported having statistically significantly (*p* < 0.001) greater subjective knowledge regarding dementia and higher levels of ageism (*p* < 0.001) compared to the Australian sample. However, they reported lower levels of stigma toward dementia in comparison with Australian participants. Finally, a larger percentage of the Israeli sample reported being acquainted with someone with dementia compared to the Australian sample (*p* < 0.001).

### 3.3. Hierarchical Regression Analyses

Table 2 displays the results for the hierarchical multiple regression. As can be observed, after controlling for gender, ethnicity, and familiarity with dementia, the best model obtained was when adding cognitive determinants (knowledge about dementia, the likelihood of developing dementia, ageism, and dementia stigma). This equation explained almost a quarter of the variance in dementia worry, with increased ageism and increased perceptions of the likelihood of developing dementia being the most important determinants associated with increased worry. Including country of residence in the model (Step 3) and interaction terms with country (Step 4) did not increase the explained variance in the dependent variable.

## 4. Discussion

Worries about getting a disease have accompanied humankind for centuries [32]. Dementia has, in recent years, become one of the most feared diseases [33], leading mostly to negative consequences such as delays in seeking help and stigmatization [2]. In an effort to reduce these consequences, researchers have investigated a variety of sociodemographic, health, psychological, and cognitive factors associated with dementia worry. However, the contribution of cultural influences has been largely ignored. To address this gap in the literature, we compared dementia worry and its determinants cross-culturally in Israel and Australia.

### 4.1. Summary of Findings

Dementia worry was found to be relatively low in both countries, as the average score was 1.78 and 1.67 for Israel and Australia, respectively, out of a 1–5 range, although it was significantly higher in Israel than in Australia, supporting our first hypothesis. Moreover, as hypothesized and similar to previous studies [5,6,7,34,35,36], positive and statistically significant associations were observed between dementia worry and knowledge of dementia, self-perceived dementia risk, ageism, and dementia stigma in both samples. However, contrary to our expectations, the interaction effects for dementia worry level by country were insignificant.

### 4.2. Cultural Differences or Not?

Upon looking broadly at our results, it is evident that understanding cultural effects is a complex process. On the one hand, the significant difference found in the level of dementia worry between the countries might indeed be associated with cultural differences, especially as previous studies have shown that more individualistic societies (i.e., Australia in this study) tend to report lower levels of worry and express less negative emotions, especially compared to others [16,18,19]. On the other hand, the levels of dementia worry were relatively similar in both countries, as were its determinants, suggesting a small impact of culture. This finding may be associated with the characteristics of the countries examined, the characteristics of the construct examined, or measurement issues.

As for the countries examined, their ranking along the individualism-collectivism dimension was reported in 2008, and there have been no additional data available since. This aspect is important, especially as Israel has been evolving over the last decades into a more individualistic and even capitalistic society [14]. Thus, Israel and Australia might be culturally more similar than it would have seemed from the 2008 data. Regarding the construct examined, as stated above, worry or fear is a basic, ubiquitous emotion that arises in the presence of a real or imagined threat [37]. As such, it is a natural, primitive human emotion that might not be affected by context.

Finally, the small effect of culture might be a consequence of the conceptualization and measurement of the dependent variable. In our study, dementia worry was assessed as an emotional reaction, despite recent developments defining it as a multidimensional construct, including a cognitive dimension in addition to an emotional one [2]. Furthermore, we did not examine other aspects of emotion such as valence or emotion arousal, which might be more sensitive to cultural differences [38]. Future studies should examine these aspects to obtain a wider and more thorough understanding of the topic under study.

The current study showed that increased ageism and an increased level of the perceived risk of developing dementia were the most important factors associated with dementia worry. These results have potentially useful implications as both can be manipulated to decrease their level or to direct their influence toward positive behaviors, such as engaging in preventive behaviors [8]. The association between perceived risk and worry is not surprising, as they reflect the cognitive and affective components of a threat, as has been found in the area of cancer worry [39]. The association with ageism might be associated with the irrational fear of aging.

### 4.3. Limitations of the Study

As with every study, there are limitations to acknowledge. First, data were collected online, potentially creating problems with the generalizability of the findings, not to mention recruitment was not available to those who are illiterate and/or not tech-savvy. The level of education for the two countries was measured differently. Hence, it was not possible to compare directly in Table 1, but our participants were highly educated and reported high levels of subjective health and financial status. In addition, people with a possible neurocognitive disorder could have taken part in this study, which may cause a problem generalizing the study findings. Future studies should screen for any dementia diagnoses and exclude these people from the study. Second, although we used structured validated measures, we relied on self-reported data. While this limitation could result in an increased social desirability bias, we believe the anonymity of the survey safeguarded the trustworthiness of the reports. Third, we failed to include several potentially important mediators or moderators of dementia worry. Based on previous studies showing the great importance of religiosity and spirituality for the study of dementia in general [40] and dementia worry in particular [41], future studies should assess these variables. Fourth, we used a cross-sectional design and are therefore unable to draw conclusions about causal relationships. Finally, we relied on the characterization of cultural aspects at the country level and did not assess cultural values at the individual level.

## 5. Conclusions

Despite these shortcomings, our study has important theoretical and practical implications. Theoretically, it expands research in the area of cultural gerontology by contributing to the understanding of dementia worry cross-culturally. It suggests that country may not be the best criterion to assess cultural differences and should be accompanied by the participants’ assessment of their cultural tendencies. In sum, at a time of globalization, differences between countries may not equal differences between cultures. Regarding dementia worry, our study stresses the importance of conceptualizing and assessing the affective and cognitive aspects of dementia worry. This notion is especially important when comparing the construct between different cultures as people from different cultures might perceive dementia worry cognitively differently but affectively similar and vice-versa. Future studies should therefore use distinct subscales separately for these dimensions in order to increase the conceptual clarity of the construct under study.

Practically, although cultural differences should be taken into account when developing intervention programs to reduce the level of dementia worry and its deleterious consequences, our findings suggest that at least in these two countries, adults reported similar levels and determinants of dementia worry. However, in order to provide a better, more culturally sensitive assessment and treatment of dementia worry, it is essential to expand the use of cross-cultural studies.

In conclusion, further research is needed to understand the importance of culture on dementia worry. Special attention should be paid to the conceptualization of the concept as well as to the operationalization of cultural differences.

## Figures and Tables

**Table 1:** Descriptive statistics of participants (% or mean (SD).

	Israeli Sample (n = 447)	Australian Sample (n = 290)	Comparison
Gender			Χ^2^(1) = 0.01, ns
% Male	49.9%	50.2%	
% Female	50.1%	49.8%	
Age	42.48 (13.10) ^a^	43.67 (14.21) ^b^	t_(581)_ = −1.15, ns
Ethnicity % Majority	77.9%	83.9%	Χ^2^_(1)_ = 4.04, *p* < 0.05
Knowledge of dementia (range: 1–5)	3.17 (0.92)	2.57 (0.89)	t_(716)_ = 8.75, *p* < 0.001
% who know someone with dementia	52.1%	36.6%	Χ^2^_(1)_ = 16.84, *p* < 0.001
Subjective assessment of health (range: 1–5)	3.99 (0.74)	3.81 (0.74)	t_(620.57)_ = 3.05, *p* < 0.01
Financial status (range: 1–5)	3.37 (0.87)	3.23 (0.90)	t_(696)_ = 2.01, *p* < 0.05
Ageism	2.05 (0.38)	1.85 (0.42)	t_(587)_ = 6.33, *p* < 0.001
Worry about developing dementia	1.78 (0.77)	1.67 (0.75)	t_(592.37)_ = 2.51, *p* < 0.05
Dementia stigma	2.44 (1.14)	3.04 (1.26)	t_(709)_ = 6.77, *p* < 0.001

^a^ Range: 18–70. ^b^ Range: 18–69. ns = not significant.

**Table 2:** Linear regression for dementia worry and its determinants.

Independent Variables	B	S.E.	β
Step 1			
Gender (female)	0.03	0.03	0.04
Minority ethnicity status	0.11	0.03	0.11 ***
Subjective health	−0.04	0.02	−0.08 *
Model statistics	*F*_(3689)_ = 11.31 ***		
	*R*^2^ = 0.05		
Step 2			
Knowledge of dementia	0.05	0.02	0.12 *
Knowing someone with dementia	0.01	0.04	0.02
Likelihood of developing dementia	0.09	0.02	0.22 ***
Ageism	0.10	0.02	0.26 ***
Dementia stigma	0.06	0.02	0.15 **
Model statistics	*F*_(8684)_ = 26.57 ***		
	*R*^2^ = 0.24		
Step 3			
Country (Australia)	−0.09	0.04	−0.12 *
Model statistics	*F*_(9683)_ = 24.04 ***		
	*R*^2^ = 0.24		
Step 4			
Knowledge of dementia × Country	0.03	0.03	0.05
Knowing someone with dementia × Country	0.09	0.06	0.08
Likelihood of developing dementia × Country (Australia)	0.01	0.03	0.01
Ageism × Country	−0.01	0.03	−0.01
Dementia stigma × Country	−0.01	0.03	−0.02
Model statistics	*F*_(14,678)_ = 15.90 ***		
	*R*^2^ = 0.25		

* *p* < 0.05, ** *p* < 0.01, *** *p* < 0.001. B = unstandardized coefficient, S.E. = standard error, β = standardized coefficient, *R*^2^ = coefficient of determination.

## Data Availability

The data presented in this study are available on request from the corresponding author.
